# CSF and plasma tau biomarkers in the Down syndrome–Alzheimer’s disease continuum

**DOI:** 10.1016/j.ebiom.2026.106370

**Published:** 2026-07-11

**Authors:** Javier Arranz, Juan Lantero-Rodríguez, Luisa Sophie Braun-Wohlfahrt, Lídia Vaqué-Alcázar, Íñigo Rodríguez-Baz, Przemysław R. Kac, Burak Arslan, Lucía Maure-Blesa, Lucía Pertierra, Laura Videla, María Carmona-Iragui, Bessy Benejam, Laura Del Hoyo Soriano, Isabel Barroeta, Susana Fernández, Alexandre Bejanin, Alberto Lleó, Nicholas J. Ashton, Henrik Zetterberg, Juan Fortea, Daniel Alcolea, Laia Montoliu-Gaya

**Affiliations:** a Sant Pau Memory Unit, IR SANT PAU, Hospital de la Santa Creu i Sant Pau, Barcelona, 08025, Spain; b Barcelona Down Medical Center, Fundació Catalana Síndrome de Down, Barcelona, 08029, Spain; c Institut de Neurociències, Universitat Autònoma de Barcelona, Barcelona, Spain; d Department of Psychiatry and Neurochemistry, Institute of Neuroscience & Physiology, The Sahlgrenska Academy at the University of Gothenburg, Mölndal, Sweden; e Centro de Investigación Biomédica en Red en Enfermedades Neurodegenerativas (CIBERNED), Madrid, 28029, Spain; f Banner Alzheimer’s Institute, Phoenix, AZ, USA; g Banner Sun Health Research Institute, Sun City, AZ, USA; h Clinical Neurochemistry Laboratory, Sahlgrenska University Hospital, Mölndal, Sweden; i Department of Neurodegenerative Disease, Queen Square Institute of Neurology, University College London, London, UK; j UK Dementia Research Institute, University College London, London, UK; k Department of Pathology and Laboratory Medicine, University of Wisconsin School of Medicine and Public Health, Madison, WI, USA; l Wisconsin Alzheimer’s Disease Research Center, University of Wisconsin School of Medicine and Public Health, University of Wisconsin-Madison, Madison, WI, USA; m Centre for Brain Research, Indian Institute of Science, Bangalore, India

**Keywords:** Down syndrome, Alzheimer’s disease, p-tau, CSF, Plasma, Staging

## Abstract

**Background:**

Nearly all individuals with Down syndrome (DS) develop Alzheimer’s disease (AD) dementia, primarily due to overexpression of the *APP* gene. Although specific cerebrospinal fluid (CSF) and plasma tau biomarkers have been investigated in DS-AD, how different tau species change in the DS-AD continuum in comparison to sporadic AD remains uncertain.

**Methods:**

In this cross-sectional study, we analysed CSF and plasma tau biomarkers in 461 samples from the DABNI and SPIN cohorts, including individuals with DS, cognitively normal euploid participants, and patients with sporadic AD. Biomarker differences were assessed using linear regression with Tukey post hoc comparisons. LOESS modelling was applied to estimate the age at which tau biomarkers became abnormal.

**Findings:**

We analysed 461 participants from the DABNI and SPIN cohorts. Both CSF and plasma tau biomarkers increased during the asymptomatic stages of DS and in euploid controls, coinciding with Aβ positivity; across the DS clinical spectrum the largest increases were observed for CSF NTA-tau (fold-change [fc] = 6.46–6.94), CSF p-tau217 (fc = 6.43–6.74) and plasma p-tau217 (fc = 4.63–6.54) (linear regression adjusted for age, sex and APOE-ε4 with Tukey post-hoc tests; all p < 0.001). During the dementia stages, CSF tau biomarkers showed only modest further increases (no CSF biomarker differed between pDS and dDS; all p ≥ 0.268), whereas plasma tau biomarkers retained a broader dynamic range across symptomatic phases (pDS vs dDS: plasma p-tau217 p = 0.001, p-tau181 p = 0.002, p-tau231 p = 0.004). Plasma p-tau217 showed the highest diagnostic accuracy, with areas under the curve (AUC) of 0.91–0.97 for biological categorisations and numerically higher values than CSF in symptomatic stages (pDS vs dDS: plasma p-tau217 AUC = 0.69 [95% CI 0.58–0.80] vs CSF p-tau217 AUC = 0.53 [95% CI 0.41–0.65]; DeLong test p = 0.019). In LOESS analyses, tau biomarkers diverged from age-matched controls in the late 30s to early 40s (e.g., plasma p-tau217 ≈ 37.3 years, CSF p-tau181 ≈ 38.1 years) and reached abnormality (+2 SD) over an approximately 20-year span between the fourth and sixth decades, outlining differential but temporally compressed increases. Finally, during symptomatic stages, tau biomarker levels remained stable in DS-AD, in contrast to sporadic AD, where levels declined with advancing age.

**Interpretation:**

These findings highlight the complementary roles of CSF and plasma tau biomarkers in tracking disease progression: CSF biomarkers capture early pathological changes, whereas plasma biomarkers more effectively reflect disease progression within symptomatic stages. Furthermore, tau biomarkers might support disease staging and monitor clinical progression in DS-AD, but with the need to adapt biomarker frameworks to this specific population.

**Funding:**

La Caixa Foundation, Instituto de Salud Carlos III, Generalitat de Catalunya, National Institute on Ageing, Wellcome Trust, Jérôme Lejeune Foundation, Medical Research Council, Alzheimer’s Association, National Institute for Health Research, EU Joint Programme–Neurodegenerative Disease Research, Alzheimer’s Society.

**Research in context**

**Evidence before this study**

Tau phosphorylation biomarkers in cerebrospinal fluid and blood have been extensively investigated in Alzheimer’s disease (AD), with a growing body of literature demonstrating their ability to reflect amyloid pathology, tau aggregation, and clinical progression across the AD continuum. More recently, several studies have proposed the use of distinct fluid tau species to biologically stage AD, capturing transitions from preclinical to symptomatic phases. These efforts have largely focused on sporadic AD populations and euploid individuals. In contrast, despite the near-universal development of AD pathology in individuals with Down syndrome (DS), biomarker-based disease staging in this population remains poorly defined. Existing studies in DS have primarily evaluated individual phosphorylated or non-phosphorylated tau markers in isolation, often with cross-sectional designs, and have not systematically compared multiple tau species across disease stages or biological compartments. As a result, it remains unclear how tau biomarkers behave longitudinally in DS-associated AD (DS-AD), how they relate to age and symptom onset, and whether staging frameworks developed for sporadic AD can be directly applied to DS.

**Added value of this study**

This study provides a comprehensive comparison of multiple cerebrospinal fluid and plasma tau biomarkers across the full disease continuum in DS-AD, benchmarked against both sporadic AD and euploid control populations. Building on the conceptual framework recently incorporated into the Alzheimer’s Association guidelines for the biological diagnosis and staging of AD, which propose the use of distinct fluid tau species to define disease stages, we evaluated the applicability of this approach in DS—a population with a markedly different genetic background and disease trajectory. Specifically, we examined (i) the dynamic trajectories of several tau biomarkers across preclinical and symptomatic stages in DS-AD relative to sporadic AD; (ii) differences in biomarker behaviour between DS individuals and age-matched euploid controls; (iii) the sequence and age-dependent timing at which individual tau markers become abnormal; and (iv) their patterns during clinically manifest disease. By directly comparing multiple tau species within and across biological fluids, this work moves beyond prior single-marker studies and provides a more integrated view of tau pathology in DS-AD.

**Implications of all the available evidence**

Collectively, the available evidence—including the findings from this study—supports the use of fluid tau biomarkers as tools for biological staging and monitoring of disease progression in DS-AD. However, our results also highlight important differences in biomarker dynamics between DS-AD and sporadic AD, indicating that staging models developed in euploid populations cannot be uncritically transferred to DS. Tailoring biomarker frameworks to the unique biological and temporal characteristics of DS-AD will be essential for improving early diagnosis, tracking disease progression, and optimising the design of preventive and disease-modifying clinical trials in this high-risk population.

## Introduction

Down syndrome (DS) is the most common genetic cause of intellectual disability. Advances in medical care have significantly increased the life expectancy of individuals with DS, which now exceeds 60 years. As a result, age-related diseases have become increasingly prevalent in this population.^1^ Among these, Alzheimer’s disease (AD) stands out as a major health concern: the lifetime risk exceeds 95% by the seventh decade of life, making AD the leading cause of death in adults with DS.^2^ The strong association between DS and AD arises from the trisomy of chromosome 21, which includes an extra copy of the amyloid precursor protein (*APP*) gene.^3^ Similar to autosomal dominant forms of AD, this genetic alteration initiates a cascade of pathogenic events: (i) overexpression of the *APP* gene, (ii) excessive production of amyloid-β (Aβ) peptides, and (iii) accumulation of Aβ into extracellular plaques.^4^ Consequently, DS is considered a genetic form of AD.^5^

Diagnosing cognitive decline due to AD in individuals with DS remains particularly challenging because of their pre-existing intellectual disability.^6^ In this context, biological diagnosis using clinically validated biomarkers—especially fluid biomarkers—could provide a valuable and accessible tool for early and accurate detection.^7^, ^8^, ^9^, ^10^

Ongoing efforts to develop fluid biomarkers for AD have yielded a wide range of soluble measurements reflecting different brain-derived proteins. Among these, tau protein biomarkers have gained particular prominence.^11^^,^^12^ Tau can be quantified through a variety of analytical approaches targeting both phosphorylated and non-phosphorylated epitopes. Using advanced platforms—including mass spectrometry and immunoassay-based techniques—several phosphorylated tau (p-tau) epitopes, such as p-tau181, p-tau205, p-tau217, p-tau231, and p-tau235, can now be reliably measured in cerebrospinal fluid (CSF) and/or plasma.^13^, ^14^, ^15^, ^16^, ^17^, ^18^, ^19^ Likewise, various assays have been developed to detect non-phosphorylated tau peptides and fragments, including NTA-tau, NT1-tau and MTBR-tau243.^20^, ^21^, ^22^, ^23^ Importantly, in both sporadic and autosomal dominant forms of AD, these newer tau biomarkers are not redundant with established CSF measures such as p-tau181 and total tau (t-tau). For instance, p-tau217 consistently demonstrates the strongest diagnostic accuracy, showing the largest mean fold changes and minimal overlap between groups.^24^^,^^25^ Furthermore, some species such as p-tau217 and p-tau231 levels are more closely associated with amyloid PET measurements, while other markers such as p-tau205 and NTA-tau display stronger correlations with tau PET.^13^^,^^26^

Building on these distinct biomarker profiles, it has been hypothesised that tau fluid biomarker alterations could enable the molecular phenotyping of individuals with AD into biological stages that reflect disease progression and severity.^27^ This concept has been incorporated into the recently published Alzheimer’s Association guidelines for the biological diagnosis and staging of AD,^28^ which propose the use of different fluid tau biomarkers to define specific biological disease stages. While this framework shows great potential for improving diagnosis and disease monitoring in sporadic AD—particularly given its slow progression and long preclinical phase^29^, ^30^, ^31^—its applicability to DS-AD remains unclear. Previous studies have studied the performance of specific isolated phosphorylated or non-phosphorylated tau biomarkers to reflect AD pathology in individuals with DS,^7^^,^^32^, ^33^, ^34^, ^35^ but never compared different tau species. In this study, we investigated: (i) how multiple CSF and plasma tau biomarkers evolve across the disease continuum in DS-AD compared with sporadic AD; (ii) how their changes in individuals with DS differ from those in euploid controls; (iii) the sequence and age-related timing of their transition to abnormal levels; and (iv) their behaviour during symptomatic disease stages.

## Methods

### Study participants and clinical classification

The studied cohort included individuals with DS, euploid cognitively normal participants, and euploid patients with sporadic AD. Participants with DS were recruited from the Down Alzheimer Barcelona Neuroimaging Initiative (DABNI), a prospective cohort established in 2014 as part of a specialised health plan at the Alzheimer Down Unit in Barcelona.^5^^,^^7^^,^^8^^,^^36^ This initiative was developed through a collaboration between the Catalan Down Syndrome Foundation and Sant Pau Hospital. The health plan involves the longitudinal evaluation of adults with DS aged ≥ 18 years, including both cognitively stable individuals, referred either for clinical assessment or due to research interest, as well as symptomatic patients undergoing diagnostic evaluation. The protocol consists of annual or semi-annual structured neurological and neuropsychological assessments conducted by experienced clinicians.

At baseline, participants were classified based on their intellectual disability level, categorised as mild, moderate, severe, or profound, following the criteria of the Diagnostic and Statistical Manual of Mental Disorders, Fifth Edition (DSM-V).^37^ This classification was based on caregivers’ reports of the individual’s best-ever level of functioning and the Kaufman Brief Intelligence Test (KBIT) Spanish version. Neurological evaluations consisted of structured anamnesis conducted with both the patient and caregiver, a physical examination, and the administration of functional scales to the caregiver. These included the Dementia Scale for People with Learning Disabilities (DLD) and the Cambridge Examination for Mental Disorders of the Elderly with Down Syndrome and Others with Intellectual Disabilities (CAMDEX-DS) interview. Additionally, neuropsychological assessments were performed using the Cambridge Cognitive Examination for Older Adults with Down Syndrome (CAMCOG-DS), adapted to the degree of Intellectual Disability. Inter-rater agreement for neuropsychological evaluations at our site has been previously assessed and demonstrated good reliability, as reported in a prior publication^38^; further details are provided in the Supplementary Methods.

The clinical classification along the AD *continuum* was initially performed independently by a neurologist and a neuropsychologist, followed by a consensus meeting to establish the final diagnosis. At each visit, participants were classified into one of three groups along the AD *continuum*. Asymptomatic DS (aDS): No clinical or neuropsychological suspicion of symptomatic AD, defined by the absence of cognitive impairment beyond the baseline Intellectual Disability level or functional decline relative to previous functioning. Prodromal AD in DS (pDS): Presence of suspected AD-related cognitive impairment, but symptoms did not fulfil criteria for dementia (no additional functional impairment). AD dementia in DS (dDS): AD dementia, characterised by cognitive impairment that significantly interfered with daily activities beyond the individual’s baseline functioning. Participants classified as sporadic AD and cognitively normal participants were recruited from the Sant Pau Initiative in Neurodegeneration (SPIN).^39^ Patients with sporadic AD included individuals with mild cognitive impairment due to AD (MCI-AD) and those in the dementia stage (i.e., AD). Diagnosis was based on the presence of cognitive disturbances identified through neuropsychological assessment, including the Clinical Dementia Rating Scale - Sum of Boxes (CDR-SoB) and functional scales such as the Interview for the Deterioration of Daily Living in Dementia (IDDD), as reported by an informant. Participants with normal cognition were volunteers that had no cognitive complaints, underwent normal physical and neuropsychological evaluations, and were confirmed to be Aβ-negative (A−) based on CSF AD biomarker assessment.^39^ Participants with symptoms (both those with DS and sporadic AD) required pathophysiological confirmation of AD (CSF A+).

### Sample collection and analysis: blood and CSF

Blood was collected immediately after lumbar puncture and simultaneously into two EDTA-K2 tubes. The samples were sent to our research laboratory, where they were processed following our previously described research protocol.^39^ In brief, blood samples were centrifuged (2000 *g* for 10 min at 4 °C) within 2 h of collection, then plasma was aliquoted into 1.5 mL polypropylene tubes and stored at −80 °C until analysis. CSF samples were collected by lumbar puncture, then centrifuged, aliquoted into 2 mL polypropylene tubes and stored at −80 °C until analysis. The detailed protocol for CSF sample collection used at our facility has been previously documented.^39^

Clinically validated CSF biomarkers, Aβ_1–42_, Aβ_1–40_, p-tau181 and t-tau, were measured during routine runs scheduled every two weeks following previously reported methods.^40^ Based on core CSF biomarker levels, participants were categorised as amyloid positive (A+, CSF Aβ_1–42_/Aβ_1–40_ ≤0.062) or negative (A−). The validation of these cutoff values has been described in prior studies.^40^ CSF p-tau231, p-tau235, p-tau205, and NTA-tau measurements were performed using in-house developed immunoassays in a Single molecule array (Simoa) HD-X platform (Quanterix) at Clinical Neurochemistry Laboratory, Sahlgrenska University Hospital, Mölndal (Sweden). Detailed information regarding assay development and validation of p-tau231, p-tau205, p-tau235 and NTA-tau has been reported elsewhere^15^^,^^17^^,^^23^^,^^41^ and more details can be found in the Supplementary Materials. CSF and plasma p-tau217 and p-tau181 were measured with commercial Simoa immunoassays (Quanterix) in the HD-X platform.

### Ethics

The study was approved by the Sant Pau Ethics Committee (Protocol code: EC/22/202/6880) following the standards for medical research in humans recommended by the 2024 revision of the Declaration of Helsinki. All participants or their legally authorised representative gave written informed consent to participate in biomarker research studies, including the use of their biological samples.

### Statistical analysis

Data normality was evaluated using the Shapiro-Wilk test. For continuous variables following a normal distribution, Student’s t-test was employed. For those not normally distributed, Kruskal-Wallis tests or log-transformed linear regression were applied. ANCOVA was conducted for group comparisons. Chi-square test was used to assess differences in categorical variables, with Fisher’s exact test applied to group comparisons with smaller sample sizes. Biomarkers were first log transformed and then z-scored with matched controls. Linear regression models adjusted by age, sex, APOE-ε4 carriership status, clinical diagnosis and ID were used to compare biomarker concentrations within groups. We used Spearman test to assess the correlation between plasma and CSF markers. Within the DS cohort, receiver operating characteristic (ROC) analyses were performed to evaluate the diagnostic performance of each biomarker. Areas under the curve (AUC) were calculated for the following contrasts: A+ versus A− defined by CSF Aβ_1–42_/Aβ_1–40_ ≤ 0.062; A+ versus A− defined by amyloid PET thresholds of ≥20, ≥25, and ≥30 Centiloids; T+ versus T− defined by CSF p-tau181 ≥ 63 pg/mL; and across clinical stages, including aDS A− versus aDS A+, aDS A+ versus pDS, aDS versus pDS, aDS versus dDS, aDS versus symptomatic DS (sDS), and pDS versus dDS. We compared the accuracy of individual markers using DeLong’s test adjusted by multiple comparisons using Bonferroni method.

We conducted individual Locally Estimated Scatterplot Smoothing (LOESS) analyses for each biomarker to determine the age at which biomarker levels begin to rise in individuals with DS. LOESS smoothing was performed using a tricubic weighting function and a span of 0.75. The LOESS curves were standardised using cognitively healthy, age-matched controls. Three complementary criteria were applied to characterise biomarker trajectories with age: (i) visual inspection of the LOESS trajectories to identify the approximate age at which biomarker levels began to separate between groups; (ii) the divergence age, defined as the point at which the 95% confidence intervals of the LOESS curves for individuals with Down syndrome and cognitively normal (CN) euploid controls no longer overlapped; and (iii) the abnormality age, defined as the first point at which the LOESS curve in DS exceeded the mean + 2 standard deviations of the corresponding biomarker in CN euploid controls. We performed combined LOESS analyses to compare (a) the sequential order in which different tau biomarkers change with age, and (b) the trajectories of these biomarkers in symptomatic individuals with DS and symptomatic individuals with sporadic AD with age. We performed sensitivity analyses with the participants that had all the biomarkers (n = 155) to assess the robustness of the results. All analyses were performed using R statistical software (version 4.2.1), with an alpha level set at 0.05.

### Role of funders

The funders of the study had no role in study design, data collection, data analysis, data interpretation, or writing of the report.

## Results

### Demographics and fluid tau biomarkers

We included a total of 461 participants from the SPIN and DABNI cohorts (Table 1). Euploid participants from the SPIN cohort were classified into three groups: CN individuals (*n* = 101), individuals with MCI-AD (*n* = 83), and those with AD dementia (*n* = 49); both MCI-AD and AD groups were Aβ-positive (A+). Participants with DS were categorised as asymptomatic Aβ negative (aDS A−, *n* = 78), aDS Aβ positive (aDS A+, *n* = 25), pDS (*n* = 49), and dDS (*n* = 76); the latter two groups were Aβ-positive.

**Table 1:** Demographic features of the participants included in the study, biomarker concentrations across diagnostic groups, CSF amyloid status and amyloid PET centiloids.

Characteristic	aDS A−, N = 78	aDS A+, N = 25	pDS, N = 49	dDS, N = 76	CN, N = 101	MCI-AD, N = 83	AD, N = 49	p-value
Age	35.9 (8.9)	45.4 (6.8)	51.5 (4.6)	51.6 (5.0)	51.8 (11.7)	71.4 (6.6)	71.6 (7.8)	<0.001
Sex (female)	28 (36%)	11 (44%)	24 (49%)	33 (43%)	69 (68%)	47 (57%)	28 (57%)	<0.001
Sex (male)	50 (64%)	14 (56%)	25 (51%)	43 (57%)	32 (32%)	36 (43%)	21 (43%)	
APOE-ε4	15 (19%)	4 (16%)	11 (23%)	17 (22%)	23 (23%)	49 (61%)	27 (55%)	<0.001
Intellectual disability								
Mild	29 (37%)	7 (28%)	9 (19%)	9 (12%)	0 (NA%)	0 (NA%)	0 (NA%)	
Moderate	40 (51%)	14 (56%)	30 (63%)	49 (64%)	0 (NA%)	0 (NA%)	0 (NA%)	
Severe/Profound	9 (12%)	4 (16%)	9 (19%)	18 (24%)	0 (NA%)	0 (NA%)	0 (NA%)	
CSF Aβ1-42 (pg/mL)	1035.4 (453.2)	613.9 (203.3)	521.0 (169.4)	475.8 (150.0)	1164.8 (386.0)	533.9 (138.1)	500.4 (150.2)	<0.001
CSF Aβ1-42/Aβ1-40	0.09 (0.02)	0.05 (0.01)	0.04 (0.01)	0.04 (0.01)	0.10 (0.01)	0.04 (0.01)	0.04 (0.01)	<0.001
CSF pTau181 (pg/mL)	28.6 (16.3)	86.2 (65.1)	161.8 (117.9)	160.3 (93.4)	33.0 (10.9)	120.5 (63.7)	132.5 (69.3)	<0.001
CSF Total Tau (pg/mL)	271.0 (136.3)	574.0 (364.3)	940.7 (553.5)	982.1 (556.4)	235.9 (81.2)	730.7 (375.5)	806.8 (362.4)	<0.001
CSF pTau205 (pg/mL)	1.1 (0.5)	3.4 (2.2)	5.3 (3.7)	6.0 (3.8)	1.5 (0.5)	5.5 (2.9)	5.7 (2.4)	<0.001
CSF NTA-Tau (pg/mL)	9.1 (8.4)	38.6 (35.6)	58.6 (49.8)	62.9 (55.6)	10.1 (6.1)	41.6 (30.4)	46.4 (31.7)	<0.001
CSF pTau235 (pg/mL)	14.6 (7.6)	28.0 (16.8)	46.5 (28.1)	47.3 (24.1)	17.4 (6.0)	45.3 (23.8)	47.4 (18.6)	<0.001
CSF pTau217 (pg/mL)	2.7 (3.0)	9.0 (6.9)	17.3 (12.9)	18.1 (11.2)	2.3 (1.5)	17.3 (12.9)	18.2 (10.3)	<0.001
CSF pTau231 (pg/mL)	95.6 (57.7)	291.2 (225.8)	514.3 (325.2)	531.7 (406.3)	139.4 (78.9)	412.4 (232.4)	553.4 (394.9)	<0.001
Plasma pTau217 (pg/mL)	0.4 (0.3)	0.9 (0.4)	1.8 (1.0)	2.5 (1.1)	0.3 (0.2)	1.3 (0.6)	1.7 (0.8)	<0.001
Plasma pTau181 (pg/mL)	11.4 (5.9)	15.5 (6.2)	22.4 (10.5)	30.8 (15.9)	12.8 (12.4)	21.4 (9.4)	22.7 (8.6)	<0.001
Plasma pTau231 (pg/mL)	8.9 (3.5)	11.0 (5.9)	16.2 (6.2)	22.0 (8.8)	11.6 (15.3)	14.4 (6.5)	16.3 (7.0)	<0.001
CSF amyloid status								<0.001
A−	78 (100%)	0 (0%)	0 (0%)	0 (0%)	101 (100%)	0 (0%)	0 (0%)	
A+	0 (0%)	25 (100%)	49 (100%)	76 (100%)	0 (0%)	83 (100%)	49 (100%)	
Centiloid	4.6 (35.4)	37.9 (32.6)	51.9 (33.4)	55.4 (18.3)	−12.8 (12.7)	NA (NA)	NA (NA)	<0.001

Data are presented as mean (SD) for continuous variables and n (%) for categorical variables. p-values were calculated using the Kruskal–Wallis test for continuous variables and Pearson’s χ^2^ test (or Fisher’s exact test) for categorical variables. aDS, asymptomatic Down syndrome (amyloid-negative or amyloid-positive subgroups); pDS, prodromal Down syndrome; dDS, Down syndrome with dementia; CN, cognitively normal euploid; MCI-AD, mild cognitive impairment due to Alzheimer’s disease; AD, Alzheimer’s disease dementia; CSF, cerebrospinal fluid; APOE-ε4, apolipoprotein E ε4 carriers.

Significant age differences were observed across groups (Kruskal–Wallis, H(6) = 320.0, p < 0.001), with asymptomatic participants with DS A− being the youngest (mean age: 35.9 ± 8.9 years) and the MCI-AD and AD groups the oldest (mean age: 71.4 ± 6.6 and 71.6 ± 7.8 years, respectively). Sex distribution also differed significantly (χ^2^(6) = 23.2, p < 0.001), with a lower proportion of females among individuals with DS compared to euploid individuals. The APOE-ε4 allele was markedly more prevalent in the MCI-AD (61%) and AD (55%) groups than in participants with DS, whose frequencies ranged from 16% to 23% (χ^2^(6) = 61.3, p < 0.001). Differences in intellectual disability severity were detected only between the aDS A− and dDS groups (χ^2^(2) = 14.4, p < 0.001), with a higher prevalence of mild intellectual disability in the aDS A− group and a greater proportion of severe/profound intellectual disability in the dDS group.

### Cross-sectional biomarker levels in CSF and plasma

**Fig. 1:**
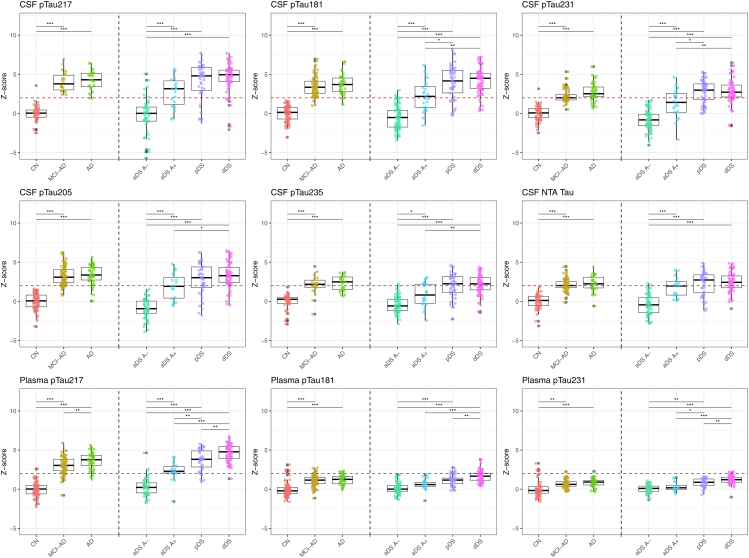
**Concentrations of tau biomarkers across groups.** CSF tau marker levels plateau during symptomatic stages in participants with DS and euploids. In contrast, plasma tau biomarkers show a stepwise increase across symptomatic stages, suggesting distinct temporal dynamics. The cutoff corresponding to two standard deviations is indicated by a red dashed line. The first row displays CSF p-tau217 — the biomarker showing the highest fold change — alongside CSF p-tau181 and p-tau231, the latter being an early biomarker in sporadic AD. The second row presents late-stage CSF biomarkers in sporadic AD, including p-tau205, p-tau235, and NTA-tau. The third row illustrates plasma biomarkers: p-tau217, p-tau181, and p-tau231. Statistical comparisons between groups were performed using linear models adjusted for age, sex, and APOE-ε4 (and additionally for intellectual disability severity in DS comparisons), with Tukey post-hoc tests. Significance levels are indicated as: ∗p < 0.05, ∗∗p < 0.01, ∗∗∗p < 0.001; comparisons without annotations correspond to non-significant results (p ≥ 0.05). Abbreviations: CN, cognitively normal; MCI-AD, mild cognitive impairment due to Alzheimer’s disease; AD, Alzheimer’s disease; aDS A−, asymptomatic Down syndrome, CSF amyloid negative; aDS A+, asymptomatic Down syndrome, CSF amyloid positive; pDS, prodromal Down syndrome; dDS, dementia Down syndrome.

Biomarker concentrations were compared across groups using linear regression models adjusted for age, sex and APOE-ε4 (and additionally for intellectual disability severity in DS comparisons), with Tukey post-hoc tests. In sporadic AD, all CSF and plasma tau biomarkers were elevated in the MCI-AD and AD groups compared with CN (all p < 0.001 except plasma p-tau231 in MCI-AD vs CN, p = 0.003) (Fig. 1 and Supplementary Table S1). The largest fold-changes (fc) between symptomatic sporadic AD groups and participants with normal cognition were observed for CSF p-tau217 (fc = 7.45–7.82), plasma p-tau217 (fc = 3.92–5.06) and CSF NTA-tau (fc = 4.11–4.58). Significant differences between the MCI-AD and AD groups were detected only for plasma p-tau217 (p = 0.005); no other biomarker differed between these groups (all p ≥ 0.062). Compared with sporadic AD, symptomatic DS-AD (mainly the dDS group) showed higher concentrations of CSF p-tau181 (p = 0.002 vs MCI-AD) as well as higher levels of all plasma tau biomarkers (p-tau217, p-tau181, and p-tau231; all p ≤ 0.045) (Supplementary Table S1).

Across the DS clinical spectrum, all tau measurements were increased in pDS and dDS compared with the aDS A− group (all p < 0.001), with the highest fold-changes observed for CSF NTA-tau (fc = 6.46–6.94), CSF p-tau217 (fc = 6.43–6.74), CSF p-tau181 (fc = 5.60–5.65), CSF p-tau231 (fc = 5.38–5.56) and plasma p-tau217 (fc = 4.63–6.54). Additionally, all CSF tau biomarker levels were higher in asymptomatic individuals aDS A+ than in aDS A− (all p ≤ 0.049), whereas among plasma markers, only p-tau217 showed a significant increase (p < 0.001; plasma p-tau181 p = 0.338, plasma p-tau231 p = 0.885). In this comparison, the largest fold-change was observed for CSF NTA-tau (fc = 4.26) and plasma p-tau217 (fc = 2.38).

When comparing the aDS A+ with pDS groups, significant differences were found for CSF p-tau181 (p = 0.028), CSF p-tau231 (p = 0.018), plasma p-tau217 (p = 0.005), and plasma p-tau231 (p = 0.029); the remaining biomarkers did not differ between these groups (all p ≥ 0.063). Comparisons between individuals with pDS and dDS revealed significant differences only in plasma biomarkers (plasma p-tau217 p = 0.001, plasma p-tau181 p = 0.002, plasma p-tau231 p = 0.004); no CSF biomarker differed between pDS and dDS (all p ≥ 0.268). Sensitivity analyses restricted to participants with complete biomarker data (n = 155) yielded consistent results within the symptomatic DS groups (Supplementary Fig. S1, Supplementary Table S2).

### Plasma and CSF biomarkers for distinguishing symptomatic stages in DS-AD

When evaluating the accuracy of all CSF and plasma tau biomarkers across biological and clinical comparisons (Supplementary Table S3), plasma p-tau217 consistently yielded the highest AUC values relative to any other CSF or plasma marker. In analyses of biological categorisations—including CSF amyloidosis, amyloid PET positivity at different thresholds, and CSF tau positivity—plasma p-tau217 showed superior performance, with AUCs ranging from 0.91 (95% CI 0.82–1.00) to 0.97 (95% CI 0.94–1.00). In the clinical domain, we examined the ability of biomarkers to discriminate between diagnostic stages (aDS A− vs. aDS A+, aDS A+ vs. pDS, aDS [combined A− and A+] vs. pDS, and pDS vs. dDS). Plasma markers, particularly p-tau217, showed numerically higher AUC values than CSF in symptomatic stages (e.g., pDS vs. dDS: plasma p-tau217 AUC = 0.69 [95% CI 0.58–0.80] vs. CSF p-tau217 AUC = 0.53 [95% CI 0.41–0.65], DeLong p = 0.019), although these differences did not remain statistically significant after correction for multiple comparisons (DeLong test with Bonferroni adjustment, threshold p < 0.0014). The results were replicated in the analyses limited only to the participants with all the tau measures (Supplementary Table S4).

### Evolution of CSF and plasma tau biomarkers in relation to age in DS-AD

**Fig. 2:**
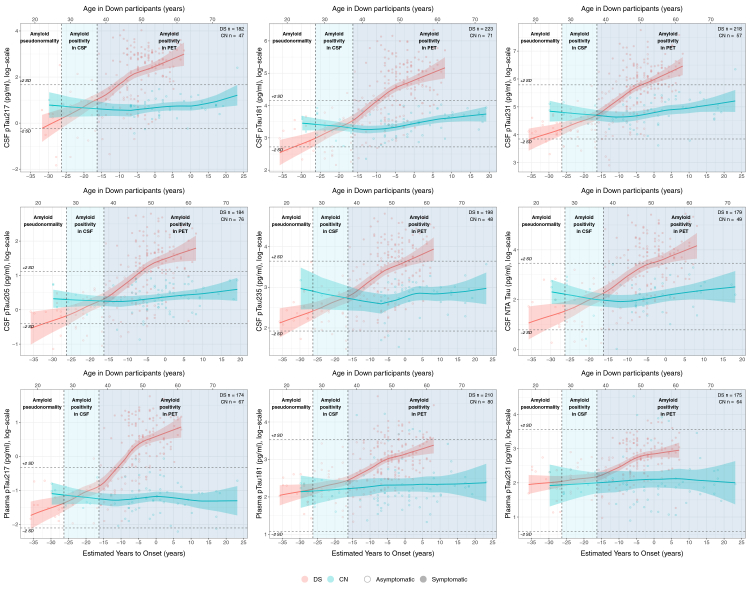
**Estimated trajectories of tau biomarkers in individuals with DS and in euploid controls.** To characterise age-related changes in tau biomarkers along the Down syndrome (DS)–Alzheimer’s continuum, we applied locally estimated scatterplot smoothing (LOESS) models to the log-transformed biomarker concentrations. For each tau biomarker, we compared the log-transformed concentrations in individuals with DS against those of age-matched cognitively normal (CN) euploid controls across the lifespan. LOESS fits were generated with locally weighted polynomial regressions, using a smoothing parameter (α) of 0.75. The shaded bands around each LOESS curve represent the 95% confidence interval of the fit. The point of divergence between DS and control trajectories was defined as the age at which these confidence bands no longer overlapped, indicating the onset of statistically relevant deviation from control biomarker levels. The horizontal black dashed lines indicate the cutoffs corresponding to ±2 standard deviations from the mean of the CN reference group, used as the threshold for biomarker abnormality. Vertical dashed lines mark established milestones along the DS–AD continuum (amyloid pseudonormality, CSF amyloid positivity, and amyloid PET positivity). For plasma biomarkers, a secondary x-axis displays the estimated years to symptom onset. Abbreviations: DS, Down Syndrome; CN, cognitively normal.

LOESS analysis for each tau biomarker in individuals with DS, relative to age-matched cognitively normal controls (Fig. 2), revealed that all biomarkers began to increase in the late 30s to early 40s. Although visual inspection of the trajectories did not reveal clear differences between groups, when using a 95% confidence interval non-overlap criterion, we identified distinct patterns of biomarker emergence. Divergence occurred at approximately 38.1 years for CSF p-tau181, 39.1 years for CSF p-tau217, 40.8 years for CSF p-tau231, 39.6 years for CSF NTA-tau, 40.9 years for CSF p-tau205 and 42.0 years for CSF p-tau235. For plasma biomarkers, the divergence was observed at around 37.3 years for plasma p-tau217, 39.9 years for plasma p-tau181 and 43.9 years for plasma p-tau231.

**Fig. 3:**
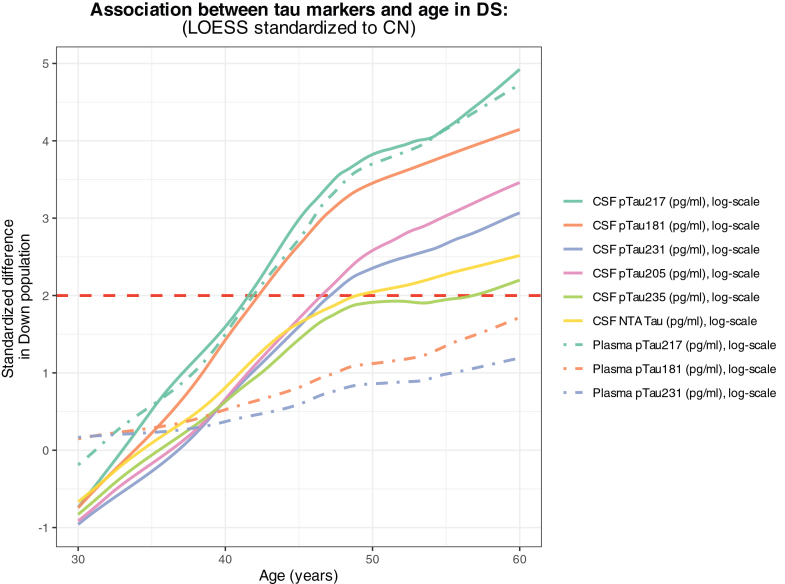
**Estimated trajectories of tau biomarkers in Down syndrome and their association with age.** To explore age-related trajectories of tau biomarkers, we applied locally estimated scatterplot smoothing (LOESS), a non-parametric regression method that fits locally weighted polynomial curves across the age continuum. First, for each participant with Down syndrome (DS), we computed the standardised difference (Z-score) of each log-transformed tau biomarker relative to age-matched euploid controls. This was achieved by subtracting the mean biomarker concentration and dividing by the standard deviation of the control group within the same age window. Thus, positive values reflect biomarker elevations, while negative values indicate lower levels compared to age-matched controls. Subsequently, we fitted LOESS curves to model the relationship between the standardised biomarker differences and age. The span (smoothing parameter α) was 0.75. Continuous lines represent CSF biomarkers, while dashed lines indicate plasma biomarkers. Corresponding plasma and CSF markers are depicted in matching colours. The cutoff of two standard deviations is indicated by a red dashed line. Abbreviations: DS, Down Syndrome; CN, cognitively normal.

We then performed a LOESS analysis combining all biomarkers and investigated the age at which each biomarker reached abnormality (defined by +2 SD from mean) (Fig. 3). We observed that tau markers became positive over a span of approximately 20 years, between the fourth and sixth decades of life. The earliest abnormalities were detected for CSF and plasma p-tau217, together with CSF p-tau181 (around 42 years). These were followed by CSF p-tau205 and CSF p-tau231 (around 47 years), CSF NTA-tau (around 49 years), and CSF p-tau235 (around 57 years). Plasma p-tau181 and p-tau231 did not reach abnormal levels during these early stages. Similar results were obtained when analyses were limited to participants with complete tau data (Supplementary Figs. S2 and S3).

### Tau biomarker levels in symptomatic phases in sporadic AD and DS-AD

**Fig. 4:**
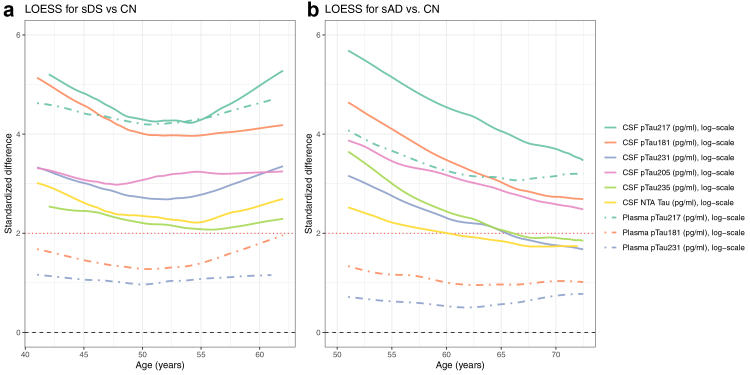
**Estimated trajectories of tau biomarkers in symptomatic stages in Down syndrome (a) and sporadic AD (b).** We applied locally estimated scatterplot smoothing (LOESS) models to the log-transformed biomarker concentrations. Continuous lines represent CSF biomarkers, while dashed lines indicate plasma biomarkers. Corresponding plasma and CSF markers are depicted in matching colours. The cutoff of two standard deviations is indicated by a red dashed line. Abbreviations: sDS, symptomatic DS; sAD, symptomatic sporadic AD; CN, cognitively normal.

Finally, we examined the relationship between CSF and plasma tau biomarkers and age in symptomatic individuals with DS-AD and individuals with sporadic AD, compared with CN euploid controls (Fig. 4, Supplementary Fig. S4). Age-related trajectories of tau biomarkers were stable in DS-AD, whereas in sporadic AD the differences in CSF and plasma tau biomarker levels relative to controls decreased with age.

## Discussion

In this study, we investigated a comprehensive panel of CSF and plasma tau biomarkers in individuals with DS and compared their changes with those observed in sporadic AD. Our results indicate that CSF and plasma tau biomarkers follow distinct trajectories across the AD continuum in both sporadic AD and DS-AD, suggesting differences in the underlying pathophysiological dynamics between central and peripheral tau processes. We further observed that tau biomarkers showed closely aligned temporal changes in their divergence from control levels, although slight differences were detected in the timing of their transition to abnormality, which occurred within a relatively compressed window. Notably, during symptomatic stages, tau biomarker levels were stable in DS-AD, in contrast to sporadic AD, where levels decreased their differences with controls with advancing age. Overall, our findings indicate that tau biomarker alterations in DS broadly mirror those observed in sporadic AD, while exhibiting distinct stage-specific patterns and magnitudes.

First, we observed that both CSF and plasma tau biomarkers increased during the asymptomatic stages of DS, coinciding with Aβ positivity. This early rise highlights their potential prognostic value in individuals at high risk of developing AD. However, CSF and plasma tau biomarkers followed distinct trajectories across the AD *continuum*, suggesting different underlying pathophysiological dynamics in each fluid. In CSF, tau biomarkers exhibited a stepwise increase from asymptomatic amyloid-negative to asymptomatic amyloid-positive individuals and further to prodromal DS, after which levels appeared to plateau at the dementia stage. A similar pattern was observed in sporadic AD, where CSF tau markers were elevated in MCI-AD and AD dementia compared with cognitively normal individuals, consistent with previous reports,^10^^,^^15^^,^^16^ but reached comparable levels in MCI-AD and AD dementia. Together, these findings suggest a deceleration in the rate of CSF tau increase once symptomatic stages are reached in both DS and sporadic AD. In contrast, plasma tau biomarkers continued to rise throughout the symptomatic stages, particularly p-tau217, showing significant increases from MCI to dementia in sporadic AD and from pDS to dDS, in line with prior findings.^42^ Notably, plasma, but not CSF, p-tau biomarker levels were higher in DS than in sporadic AD. Collectively, these results highlight the complementary roles of CSF and plasma tau biomarkers in tracking AD pathology: CSF biomarkers primarily capture early pathological changes, whereas plasma biomarkers more sensitively reflect disease progression during symptomatic stages. One possible explanation is that CSF tau biomarkers may reach a plateau earlier in the disease course due to saturation of central pathological processes, whereas plasma tau may reflect similar changes with a temporal delay. However, peripheral factors such as clearance mechanisms or systemic metabolism may also contribute to the observed variability in plasma concentrations. Among plasma biomarkers, p-tau217 consistently demonstrated the highest accuracy for detecting AD pathology in both DS and sporadic AD, as previously reported.^13^^,^^14^^,^^25^^,^^43^ In addition, we observed that plasma p-tau217 emerges early and crosses the threshold of abnormality early along the DS-AD continuum. In contrast, plasma p-tau181 and p-tau231 did not exceed the 2-SD abnormality threshold with disease progression, suggesting limited sensitivity to central nervous system pathology^44^ and reduced utility for staging AD in blood.^30^

Prior studies in sporadic and autosomal dominant AD have suggested a largely sequential cascade of changes in fluid tau biomarkers.^13^^,^^18^^,^^29^^,^^30^ These reports converge on several key biomarker milestones: (i) p-tau181 and p-tau217 emerge earliest, (ii) p-tau205 increases later, closer to the onset of tau PET positivity, and (iii) non-phosphorylated tau species—including t-tau, MTBR-tau243, and NTA-tau—are the last to reach abnormal levels. In the present study, we observed a broadly similar sequence in DS-AD. Specifically, p-tau217 and p-tau181 showed the earliest changes, followed by p-tau205 and NTA-tau, with p-tau235 emerging last. Notably, the late appearance of p-tau235—even after non-phosphorylated tau forms—suggests that certain phosphorylation events occur very late in the disease course, potentially only once tau becomes highly phosphorylated, as previously proposed.^45^ Overall, this pattern resembles findings from studies in sporadic and autosomal dominant AD^18^^,^^29^^,^^30^ motivating the concept of staging AD using biofluid tau measurements.^46^ However, the temporal separation between biomarkers appears more compressed in DS. Consistent with this interpretation, AD pathology progression in DS has been shown to be accelerated relative to sporadic AD, as demonstrated by both amyloid and tau PET studies.^47^^,^^48^

Finally, we observed that during symptomatic stages, tau levels in individuals with DS-AD dementia were similar across ages in both CSF and plasma. In contrast, in euploid individuals, CSF tau levels tended to converge with advancing age, narrowing the difference between individuals with normal cognition and those with sporadic AD. Previous studies have reported a decline in fluid p-tau biomarker levels over time in sporadic AD.^49^ More recently, two independent studies have demonstrated a reduction in tau PET during symptomatic stages of sporadic AD, particularly at the dementia stage.^50^^,^^51^ The observation that tau biomarkers decrease in late stages of sporadic AD but remain stable in DS-AD suggests distinct underlying pathophysiological mechanisms. In DS-AD, the triplication of the APP gene may lead to sustained Aβ overproduction, potentially maintaining elevated tau pathology even in advanced disease stages.

While many pathological features are shared among DS-AD, autosomal dominant AD, and sporadic AD, DS-AD exhibits distinct pathological characteristics attributable to trisomy 21. These include more pronounced alterations in immune-related proteins, extracellular matrix pathways, and plasma proteins, likely reflecting blood–brain barrier dysfunction. Importantly, such changes are detectable in young adults with DS before the emergence of amyloid-β or tau pathology, suggesting that they are directly linked to trisomy 21 and may constitute early risk factors for DS-AD.^52^ Likely also as a consequence of trisomy 21, the temporal sequence of AD-related pathological alterations in DS appears to be more predetermined, time-compressed, and rapidly progressive,^48^ potentially reflecting a more continuous disease process with a limited or absent plateau phase. Chronic neuroinflammation associated with DS may also contribute to variability in plasma tau concentrations.^53^ In contrast, vascular pathology is generally less prominent in DS than in sporadic AD, making vascular contributions to plasma tau variability less likely.^54^ Previous studies have also reported higher plasma tau levels and different diagnostic thresholds in individuals with DS.^33^ Collectively, these observations underscore the need to specifically characterise AD biomarker dynamics in DS and to adapt diagnostic and staging frameworks accordingly.

The main strengths of this study include the comprehensive assessment of multiple tau biomarkers—including the use of in-house assays—and the analysis of two independent, well-characterised cohorts. Nevertheless, several limitations should be acknowledged. First, the relatively small sample size and incomplete biomarker availability across participants limited our ability to establish a staging framework for DS-associated AD. Second, the estimated ages at which tau biomarkers diverged from control levels and transitioned to abnormality are model-dependent estimates that are highly sensitive to the choice of control group and to the dynamic range of the assays used. These estimates should therefore be interpreted with caution and should not be considered definitive biological thresholds or reference values. Third, we observed a later emergence of p-tau231 in DS-AD compared with reports in sporadic AD based on immunoassay-based techniques^24^^,^^41^^,^^55^; however, this is not the case when comparing with mass spectrometry–based measurements.^30^ Fourth, sex distribution differed slightly between groups, with a higher proportion of females among euploid participants. Although our analyses were statistically adjusted for sex, previous studies suggest that sex may influence tau biomarker levels and trajectories. Therefore, residual effects related to sex differences between cognitively normal euploid participants and individuals with DS cannot be entirely excluded and should be considered when interpreting the results.^56^ Fifth, the absence of longitudinal data limited our ability to model individual biomarker trajectories and to assess their utility for disease monitoring over time. Sixth, participants were predominantly White/Caucasian, reflecting the demographic characteristics of the population served by our recruitment sites. This limited ethnic diversity may restrict the generalisability of the findings to other racial or ethnic groups. Finally, the diagnosis of cognitive decline in individuals with DS—particularly during prodromal stages—remains challenging and may have affected the clinical classification of asymptomatic and symptomatic groups.

In conclusion, the stabilisation of CSF tau levels during early symptomatic stages, together with the progressive and stepwise rise of plasma tau biomarkers across clinical progression, indicates distinct temporal dynamics between central and peripheral compartments. Moreover, in DS-AD, tau biomarkers reach sequential points of abnormality across the lifespan in a manner broadly comparable to sporadic AD, although within a more compressed time window. Finally, during symptomatic stages, tau biomarker levels appear to remain stable with age in DS-AD, whereas they decline in sporadic AD in relation to controls. Taken together, these findings support the utility of tau biomarkers for disease staging and monitoring clinical progression in DS-AD, while underscoring the need to adapt biomarker frameworks developed in sporadic AD to the specific biological context of DS.

## Contributors

JA, JLR, JF, DA and LMG created the concept and design. Data acquisition was performed by JLR, LSBW and LMG. JA and JLR accessed and verified the underlying data, and performed data analysis. LSBW, LVA, IRB, PRK, BA, LMB, LP, LV, MCI, BB, LDHS, IB, SF, AB, AL, NJA, HZ and JF contributed to the interpretation of data. JA, JLR, DA and LMG drafted the manuscript, and LSBW, LVA, IRB, PRK, BA, LMB, LP, LV, MCI, BB, LDHS, IB, SF, AB, AL, NJA, HZ and JF revised. All authors read and approved the final manuscript.

## Data sharing statement

Anonymised data will be shared by request from a qualified academic investigator for the sole purpose of replicating procedures and results presented in the article and as long as data transfer is in agreement with EU legislation on the general data protection regulation and decisions by the Ethical Review Board, which should be regulated in a material transfer agreement.

## Declaration of interests

JA has received personal fees for service on the advisory boards, speaker honoraria or educational activities from Esteve, Lilly, and Roche diagnostics, and travel support from Fujirebio, and grants from Rio Hortega Grant Instituto de Salud Carlos III. LMB is supported by Instituto de Salud Carlos III through the Río Hortega Fellowship “CM23/00291” and cofounded by the European Union. JF has received consulting/honoraria fees from Lundbeck, Ionis, AC Immune Roche, Esteve, Biogen, Laboratorios Carnot, Adamed, LMI, Eisai, and Lilly; and has participated on advisory boards for AC Immune, Alzheon, Zambon, Roche, Lilly, Eisai, Perha and Pri; he is co-owner of the patent WO2019175379 A1 Markers of synaptopathy in neurodegenerative disease and has served in the following society boards: Spanish Neurological Society, T21 Research Society, Lumind Foundation, Jérôme-Lejeune Foundation, Alzheimer’s Association, Health Research Board (HRB), Dementia Trials Ireland, European commission, National Institutes of Health, and Instituto de Salud Carlos III; he has received laboratory material from Life Molecular Imaging (LMI). MCI has received personal fees for service on the advisory boards, speaker honoraria or educational activities from Esteve, Lilly, Neuraxpharm, and Roche; and grants to her institution from Instituto de Salud Carlos III, Spain (PI18/00335, PI22/00785, ICI23/00032), Alzheimer’s Association (AARG-22-973966); MCI is Co-chair Science & Society Committee of the T21RS (unpaid). IRB has received travel support from Esteve, Nutricia and Almirall, is supported by Instituto de Salud Carlos III through the Río Hortega Fellowship “CM22/00052” and co-funded by the European Union, Alzheimer’s Association (AACSF-25-1486364). IB has received fees from educational events from Adium. NJA has received consulting fees from Abbvie, Athria, ImaginationLand LLC, MapLight, Therapeutics, SpearBio, Neurogen Biomarking, Quanterix, TauRx, Eli-Lily, Roche Dx, Beckman, Coulter, Janssen, Bristol Myers Squibb, ImmunoBrain; honoraria from Alamar Biosciences, Biogen, Eli-Lilly, Quanterix, VJDementia, Beckman Coulter; royalties from the patent application No.: PCT/US2024/037834 (WSGR Docket No. 58484-709.601), Methods for Remote Blood Collection, Extraction and Analysis of Neuro Biomarkers; and has participated in advisory boards for Biogen, Bristol Myers Squibb, New Amsterdam, Janssen, Roche and TauRx. AL reported receiving personal fees for service on advisory boards, or speaker honoraria from Beckman-Coulter, Eisai, Esteve, Fujirebio-Europe, NovoNordisk, Roche, Grifols S.A., Lilly, and Global Learning Collaborative; and grant to his institution from Fondo de Investigaciones Sanitario, Carlos III, Health Institute Research grant, Alzheimer’s Association Research grant, Novo Nordisk Research grant, Ajuntament de Barcelona, in collaboration with La Caixa Foundation. AL is co-author of a patent for markers of synaptopathy in neurodegenerative disease (licensed to ADx, EPI8382175.0) and on antibodies for amyloid precursor, methods and uses thereof European priority (N°EP25382226). HZ has served at scientific advisory boards and/or as a consultant for Abbvie, Acumen, Alector, Alzinova, ALZpath, Amylyx, Annexon, Apellis, Artery Therapeutics, AZTherapies, Cognito Therapeutics, CogRx, Denali, Eisai, Enigma, LabCorp, Merck Sharp & Dohme, Merry Life, Nervgen, Novo Nordisk, Optoceutics, Passage Bio, Pinteon Therapeutics, Prothena, Quanterix, Red Abbey Labs, reMYND, Roche, Samumed, ScandiBio Therapeutics AB, Siemens Healthineers, Triplet Therapeutics, and Wave, has given lectures sponsored by Alzecure, BioArctic, Biogen, Cellectricon, Fujirebio, LabCorp, Lilly, Novo Nordisk, Oy Medix Biochemica AB, Roche, and WebMD, is a co-founder of Brain Biomarker Solutions in Gothenburg AB (BBS), which is a part of the GU Ventures Incubator Program, is chair of the Alzheimer’s Association Global Biomarker Standardisation Consortium and chair of the IFCC WG-BND, and is a shareholder of CERimmune Therapeutics (outside submitted work). DA has received consulting fees from Grifols S.A., Lilly, Fujirebio-Europe, Roche Diagnostics, and Schwabe; honoraria from Fujirebio-Europe, Roche Diagnostics, Nutricia, Krka Farmacéutica SL, Zambon SAU, Esteve Pharmaceuticals, Neuraxpharm, Alter, and Lilly; support for attending meetings from Fujirebio-Europe, Lilly, Nutricia and Novo Nordisk; participated on Data Safety Monitoring Board or Advisory Board for Grifols S.A., Lilly, Fujirebio-Europe, Roche Diagnostics; and is co-inventor in the patent WO2019175379 A1 Markers of synaptopathy in neurodegenerative diseases. LMG has received consultancy/speaker fees from Quaterix, Esteve and Neurocode, and royalties from the patent application No.: PCT/US2024/037834 (WSGR Docket No. 58484-709.601), Methods for Remote Blood Collection, Extraction and Analysis of Neuro Biomarkers; and has one pending patent application: 63/514,607, Title of Invention P-TAU IMMUNOASSAY. Date of application 07/20/2023. All the other authors declare no conflicts of interest.
