# Supplementary material for: CSF and plasma tau biomarkers in the Down syndrome–Alzheimer’s disease continuum
Source: eBioMedicine. 2026 Jul 11;130:106370. doi: 10.1016/j.ebiom.2026.106370 (PMC13382054; doi:10.1016/j.ebiom.2026.106370)
Supplement: Supplementary Methods, Figs. S1–S4 and Tables S1–S3 [file mmc1.docx]

**CSF AND PLASMA TAU BIOMARKERS IN THE DOWN SYNDROME ALZHEIMER´S DISEASE CONTINUUM**

Javier Arranz*^1,3^, Juan Lantero-Rodríguez*^4^, Luisa Sophie Braun-Wohlfahrt^4^, Lídia Vaqué-Alcázar^1^, Íñigo Rodríguez-Baz^1,5^, Przemysław R Kac^4^, Burak Arslan^4^, Lucía Maure-Blesa^1,3^, Lucía Pertierra^1,3^, Laura Videla^1,2^, María Carmona-Iragui^1,2,3,5^, Bessy Benejam^1,2^, Laura Del Hoyo Soriano^1,5^, Isabel Barroeta^1,5^, Susana Fernández^2^, Alexandre Bejanin^1,5^, Alberto Lleó^1,5^, Nicholas J Ashton^4,6,7^, [Henrik Zetterberg](https://pubmed.ncbi.nlm.nih.gov/?sort=pubdate&size=100&term=Zetterberg+H&cauthor_id=40595720)^4,8,9,10,11,12,13^, Juan Fortea^1, 2, 3,5^, Daniel Alcolea^1,3^**& Laia Montoliu-Gaya**^§4,1^.

* Javier Arranz and Juan Lantero-Rodríguez share first position.

** Daniel Alcolea and Laia Montoliu-Gaya share last position.

§ correspondence to laia.montoliu.gaya@gu.se

**Affiliations:**

^1^ Sant Pau Memory Unit, IR SANT PAU, Hospital de la Santa Creu i Sant Pau, Barcelona, 08025, Spain.

^2^ Barcelona Down Medical Center, Fundació Catalana Síndrome de Down, Barcelona, 08029, Spain.

^3^ Institut de Neurociències, Universitat Autònoma de Barcelona, Barcelona, Spain

^4^ Department of Psychiatry and Neurochemistry, Institute of Neuroscience & Physiology, The Sahlgrenska Academy at the University of Gothenburg, Mölndal, Sweden

^5^ Centro de Investigación Biomédica en Red en Enfermedades Neurodegenerativas (CIBERNED), Madrid, 28029, Spain.

^6^ Banner Alzheimer's Institute, Phoenix, AZ, USA. nicholas.ashton@gu.se.

^7^ Banner Sun Health Research Institute, Sun City, AZ, USA. nicholas.ashton@gu.se.

^8^ Clinical Neurochemistry Laboratory, Sahlgrenska University Hospital, Mölndal, Sweden

^9^ Department of Neurodegenerative Disease, Queen Square Institute of Neurology, University College London, London, UK

^10^ UK Dementia Research Institute, University College London, London, UK

^11^ Department of Pathology and Laboratory Medicine, University of Wisconsin School of Medicine and Public Health, Madison, WI, USA

^12^ Wisconsin Alzheimer’s Disease Research Center, University of Wisconsin School of Medicine and Public Health, University of Wisconsin-Madison, Madison, WI, USA

^13^ Centre for Brain Research, Indian Institute of Science, Bangalore, India

**Supplementary Material**

[Supplementary Methods 3](#_Toc216978678)

[Assay development and validation of CSF p-tau205 and NTA-tau 3](#_Toc216978679)

[Supplementary Tables 4](#_Toc216978680)

[Supplementary Table 1. Fold-changes of tau markers 4](#_Toc216978681)

[Supplementary Table 2. Diagnostic accuracy of CSF and plasma biomarkers across biological and clinical conditions. 5](#_Toc216978682)

[Supplementary Table 3. Diagnostic accuracy of CSF and plasma biomarkers across biological and clinical conditions restricted to participants with all the markers (Sensitivity analysis). 8](#_Toc216978683)

[Supplementary Figures 11](#_Toc216978684)

[Supplementary Figure 1. Concentrations of tau markers in CSF and plasma 11](#_Toc216978685)

[Supplementary Figure 2. Estimated trajectories of tau biomarkers in Down syndrome (only participants with all Tau markers available) 12](#_Toc216978686)

[Supplementary Figure 3. Estimated trajectories of tau biomarkers in Down syndrome and their association with age (only participants with all Tau markers available) 13](#_Toc216978687)

[Supplementary Figure 4. Estimated trajectories of tau biomarkers in symptomatic stages in Down syndrome (a) and sporadic AD (b). Only participants with all Tau markers available. 14](#_Toc216978688)

# Supplementary Methods

## Assay development and validation of CSF p-tau205 and NTA-tau

In brief, CSF p-tau235 and p-tau205 assays are comprised by a rabbit polyclonal antibody directed against phosphorylated tau at serine 235 and threonine 205 (respectively), conjugated to paramagnetic beads and used as capture antibody. CSF NTA-tau assay uses a mouse monoclonal antibody HT7 directed against tau mid-region, conjugated to paramagnetic beads and used as capture antibody. All assays used biotinylated mouse monoclonal Tau13 for detection. Eight-point calibration curves were generated using commercially available GSK-3β phosphorylated recombinant full-length Tau411 (for CSF p-tau235 and p-tau205 assays) and commercially available non-phosphorylated recombinant full-length Tau411 (for CSF NTA-tau assay). Calibration curve was run always in duplicates. Randomized CSF samples were thawed for 45 minutes at room temperature and vortexed immediately after. Prior to plating, samples were then diluted using commercially available Tau2.0 assay diluent (Quanterix). All plates contained internal quality control samples, which were run in duplicates before and after the samples. Repeatability and intermediate precision in the cohort was below 15%.

**Sample size calculation**

The sample size was determined by the availability of participants within the SPIN and DABNI cohorts. The resulting sample (n = 461) is comparable to or larger than previous biomarker studies in Down syndrome–associated and sporadic Alzheimer’s disease populations.

**Inter-rater agreement of neuropsychological assessments in the DABNI cohort**

Neuropsychological evaluations in participants with Down syndrome (DABNI cohort) were performed by experienced neuropsychologists using the Cambridge Cognitive Examination for Older Adults with Down’s Syndrome (CAMCOG‐DS) and complementary tests, administered with validated language- and culture-specific versions. Inter-rater agreement of these cognitive assessments was formally evaluated in a recent dual-center study including 673 adults with Down syndrome of mild to moderate intellectual disability across different stages of Alzheimer’s disease (Del Hoyo Soriano et al., Alzheimer’s & Dementia 2025;21:e70307). Inter-rater agreement for intellectual disability level classification within site was 95%, supporting the consistency of the cognitive assessments underlying the diagnostic categorisation used in the present study. The same study highlighted that inter-rater agreement between sites was lower (60%), underscoring the importance of standardised intellectual disability classification when applying site-specific cognitive cut-offs across centres, a consideration relevant for the interpretation of cross-cohort comparisons in Down syndrome research.

# Supplementary Tables

## Supplementary Table 1. Fold-changes of tau markers
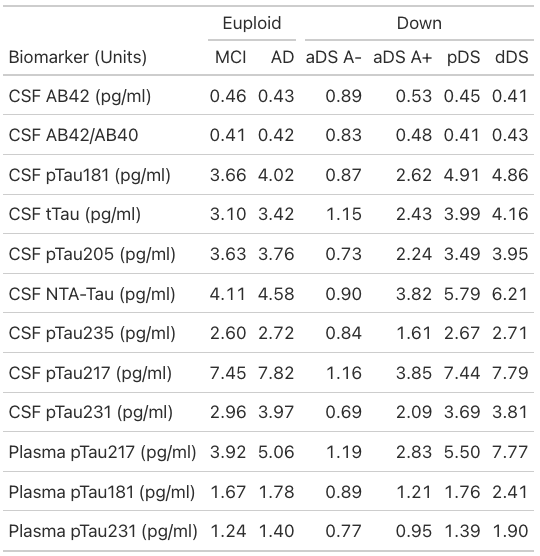

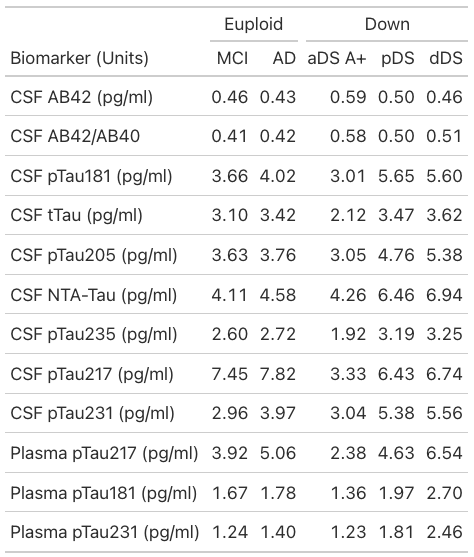


Tables present the fold changes of tau markers, calculated A) relative to CN for sporadic AD and aDS A- for DS, and B) relative to CN for both sporadic AD and DS. A similar pattern is observed in both tables, where fold changes are higher in CSF biomarkers and plasma pTau217 during the prodromal phases of sporadic AD and DSAD. Subsequently, when comparing to its control group (A) mainly plasma biomarkers continue to increase significantly from prodromal to dementia stages in Down syndrome. When comparing DS to CN (B), we find similar results.

Abbreviations: CN, cognitively normal. MCI-AD, mild cognitive impairment Alzheimer’s Disease. AD, Alzheimer’s Disease. aDS A-, asymptomatic Down Syndrome CSF Amyloid negative. aDS A+, asymptomatic Down Syndrome CSF Amyloid positive. pDS, prodromal Down Syndrome. dDS, dementia Down Syndrome.

**
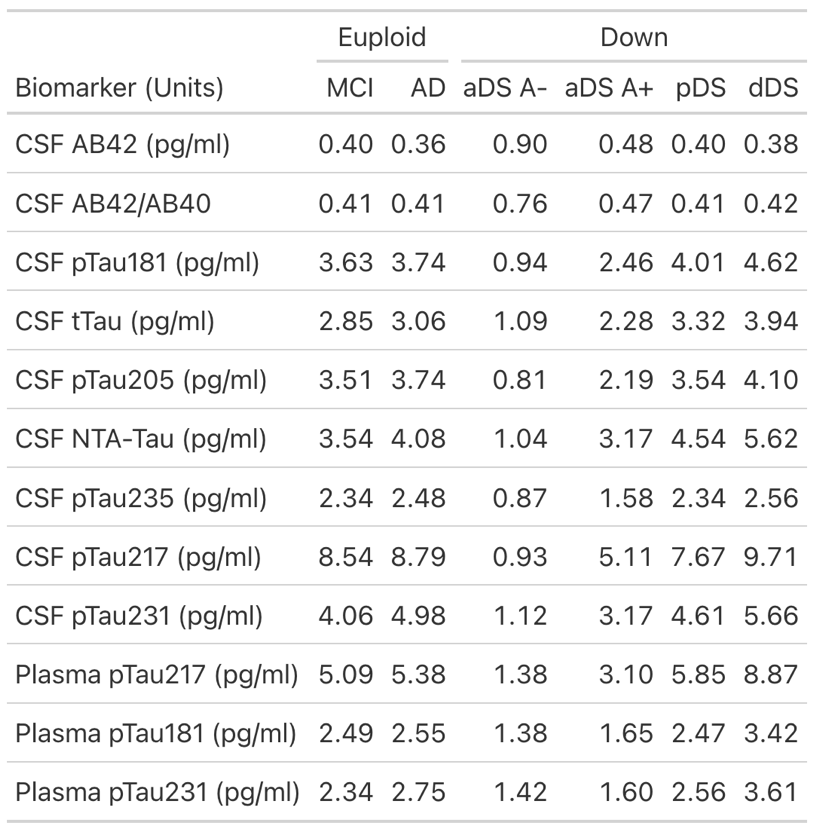

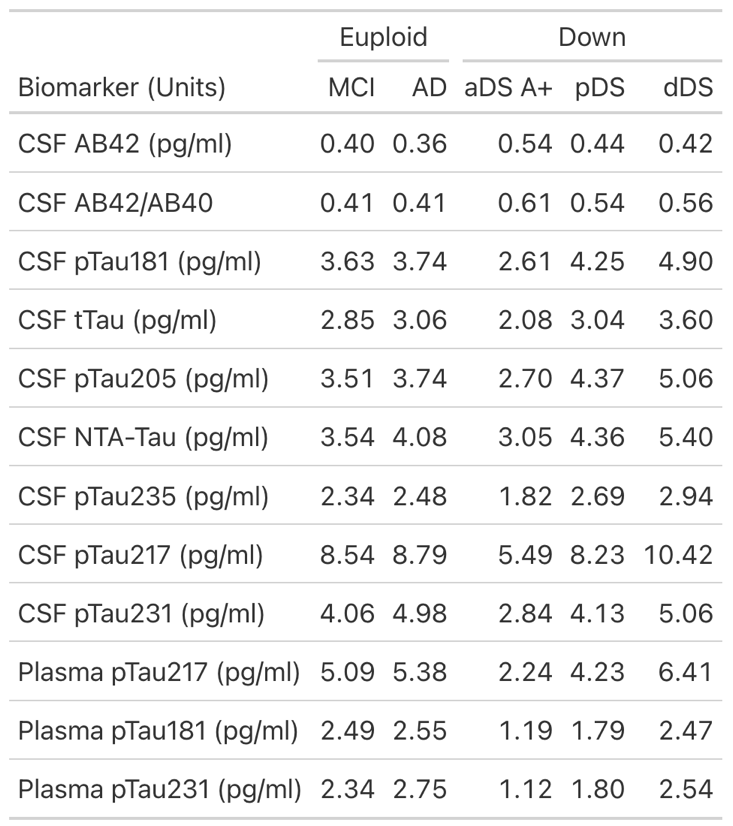
Supplementary Table 2. Fold-changes of tau markers restricted to participants with all the markers (Sensitivity analysis)**

Tables present the fold changes of tau markers, calculated A) relative to CN for sporadic AD and aDS A- for DS, and B) relative to CN for both sporadic AD and DS. A similar pattern is observed in both tables, where fold changes are higher in CSF biomarkers and plasma pTau217 during the prodromal phases of sporadic AD and DSAD. Subsequently, when comparing to its control group (A) mainly plasma biomarkers continue to increase significantly from prodromal to dementia stages in Down syndrome. When comparing DS to CN (B), we find similar results.

Abbreviations: CN, cognitively normal. MCI-AD, mild cognitive impairment Alzheimer’s Disease. AD, Alzheimer’s Disease. aDS A-, asymptomatic Down Syndrome CSF Amyloid negative. aDS A+, asymptomatic Down Syndrome CSF Amyloid positive. pDS, prodromal Down Syndrome. dDS, dementia Down Syndrome.

## Supplementary Table 3. Diagnostic accuracy of CSF and plasma biomarkers across biological and clinical conditions.

| Comparison | Marker | AUC | CI_lower | CI_upper | Controls | Cases |
| --- | --- | --- | --- | --- | --- | --- |
| ASTATUS | CSF_pTau217 | 0.9102071 | 0.86704899 | 0.95336521 | 52 | 130 |
| ASTATUS | CSF_pTau181 | 0.95143213 | 0.9273346 | 0.97552966 | 77 | 146 |
| ASTATUS | CSF_pTau231 | 0.94538288 | 0.91736068 | 0.97340509 | 74 | 144 |
| ASTATUS | CSF_pTau205 | 0.94542373 | 0.91609939 | 0.97474806 | 59 | 125 |
| ASTATUS | CSF_pTau235 | 0.88854454 | 0.84391052 | 0.93317856 | 66 | 132 |
| ASTATUS | CSF_NTA_Tau | 0.91806783 | 0.87926854 | 0.95686712 | 58 | 121 |
| ASTATUS | pTau217p_S | 0.96933573 | 0.94123357 | 0.9974379 | 57 | 117 |
| ASTATUS | pTau181p | 0.88280008 | 0.83426942 | 0.93133074 | 74 | 136 |
| ASTATUS | pTau231p | 0.85819298 | 0.80488506 | 0.91150089 | 62 | 113 |
| TSTATUS | CSF_pTau217 | 0.95956386 | 0.92954469 | 0.98958303 | 75 | 107 |
| TSTATUS | CSF_pTau181 | 1 | 1 | 1 | 103 | 120 |
| TSTATUS | CSF_pTau231 | 0.97720339 | 0.95573868 | 0.9986681 | 100 | 118 |
| TSTATUS | CSF_pTau205 | 0.9760119 | 0.95400968 | 0.99801413 | 84 | 100 |
| TSTATUS | CSF_pTau235 | 0.97520576 | 0.95456121 | 0.99585031 | 90 | 108 |
| TSTATUS | CSF_NTA_Tau | 0.97334674 | 0.95103577 | 0.99565771 | 82 | 97 |
| TSTATUS | pTau217p_S | 0.92267287 | 0.88345598 | 0.96188977 | 80 | 94 |
| TSTATUS | pTau181p | 0.87422558 | 0.82731952 | 0.92113165 | 98 | 112 |
| TSTATUS | pTau231p | 0.83588235 | 0.7745007 | 0.89726401 | 85 | 90 |
| CENT20 | CSF_pTau217 | 0.76481481 | 0.62097442 | 0.90865521 | 20 | 27 |
| CENT20 | CSF_pTau181 | 0.85972851 | 0.76709524 | 0.95236177 | 26 | 34 |
| CENT20 | CSF_pTau231 | 0.81923077 | 0.70196362 | 0.93649792 | 26 | 30 |
| CENT20 | CSF_pTau205 | 0.8482906 | 0.72507637 | 0.97150483 | 18 | 26 |
| CENT20 | CSF_pTau235 | 0.77175698 | 0.63338155 | 0.91013241 | 21 | 29 |
| CENT20 | CSF_NTA_Tau | 0.80235294 | 0.65529349 | 0.94941239 | 17 | 25 |
| CENT20 | pTau217p_S | 0.91776316 | 0.82449257 | 1 | 19 | 24 |
| CENT20 | pTau181p | 0.86482759 | 0.76894174 | 0.96071343 | 25 | 29 |
| CENT20 | pTau231p | 0.84773663 | 0.72510245 | 0.9703708 | 18 | 27 |
| CENT25 | CSF_pTau217 | 0.74 | 0.59538395 | 0.88461605 | 22 | 25 |
| CENT25 | CSF_pTau181 | 0.83258929 | 0.73278819 | 0.93239039 | 28 | 32 |
| CENT25 | CSF_pTau231 | 0.78826531 | 0.66746006 | 0.90907055 | 28 | 28 |
| CENT25 | CSF_pTau205 | 0.83368421 | 0.70997775 | 0.95739067 | 19 | 25 |
| CENT25 | CSF_pTau235 | 0.74396135 | 0.60397799 | 0.88394472 | 23 | 27 |
| CENT25 | CSF_NTA_Tau | 0.76388889 | 0.60644108 | 0.9213367 | 18 | 24 |
| CENT25 | pTau217p_S | 0.91413043 | 0.81924978 | 1 | 20 | 23 |
| CENT25 | pTau181p | 0.8340192 | 0.72849385 | 0.93954456 | 27 | 27 |
| CENT25 | pTau231p | 0.804 | 0.67278484 | 0.93521516 | 20 | 25 |
| CENT30 | CSF_pTau217 | 0.74181818 | 0.59483747 | 0.8887989 | 25 | 22 |
| CENT30 | CSF_pTau181 | 0.82924107 | 0.72763084 | 0.9308513 | 32 | 28 |
| CENT30 | CSF_pTau231 | 0.78333333 | 0.66330653 | 0.90336013 | 30 | 26 |
| CENT30 | CSF_pTau205 | 0.8088843 | 0.68271188 | 0.93505671 | 22 | 22 |
| CENT30 | CSF_pTau235 | 0.73557692 | 0.59507181 | 0.87608204 | 26 | 24 |
| CENT30 | CSF_NTA_Tau | 0.74376417 | 0.59272775 | 0.8948006 | 21 | 21 |
| CENT30 | pTau217p_S | 0.91883117 | 0.82352349 | 1 | 21 | 22 |
| CENT30 | pTau181p | 0.87448276 | 0.7856033 | 0.96336222 | 29 | 25 |
| CENT30 | pTau231p | 0.82539683 | 0.70187293 | 0.94892072 | 21 | 24 |
| aDS_pDS | CSF_pTau217 | 0.83735521 | 0.75675092 | 0.9179595 | 74 | 42 |
| aDS_pDS | CSF_pTau181 | 0.89367347 | 0.84073667 | 0.94661027 | 100 | 49 |
| aDS_pDS | CSF_pTau231 | 0.90360399 | 0.85634449 | 0.9508635 | 98 | 47 |
| aDS_pDS | CSF_pTau205 | 0.85318949 | 0.77687429 | 0.92950469 | 78 | 41 |
| aDS_pDS | CSF_pTau235 | 0.84220041 | 0.76735343 | 0.9170474 | 88 | 44 |
| aDS_pDS | CSF_NTA_Tau | 0.82922078 | 0.74994276 | 0.9084988 | 77 | 40 |
| aDS_pDS | pTau217p_S | 0.91962963 | 0.87023737 | 0.96902189 | 75 | 36 |
| aDS_pDS | pTau181p | 0.82492898 | 0.75155086 | 0.89830709 | 96 | 44 |
| aDS_pDS | pTau231p | 0.82051282 | 0.73573987 | 0.90528578 | 78 | 37 |
| aDS_dDS | CSF_pTau217 | 0.88462326 | 0.82568842 | 0.9435581 | 74 | 66 |
| aDS_dDS | CSF_pTau181 | 0.93094595 | 0.89372806 | 0.96816383 | 100 | 74 |
| aDS_dDS | CSF_pTau231 | 0.91137825 | 0.86506242 | 0.95769408 | 98 | 73 |
| aDS_dDS | CSF_pTau205 | 0.91755424 | 0.87140968 | 0.9636988 | 78 | 65 |
| aDS_dDS | CSF_pTau235 | 0.88464187 | 0.83038184 | 0.93890191 | 88 | 66 |
| aDS_dDS | CSF_NTA_Tau | 0.87327189 | 0.81505897 | 0.9314848 | 77 | 62 |
| aDS_dDS | pTau217p_S | 0.9762963 | 0.95568091 | 0.99691169 | 75 | 63 |
| aDS_dDS | pTau181p | 0.92894345 | 0.89249525 | 0.96539166 | 96 | 70 |
| aDS_dDS | pTau231p | 0.93076923 | 0.88620218 | 0.97533628 | 78 | 60 |
| aDS_sDS | CSF_pTau217 | 0.86624124 | 0.81196345 | 0.92051904 | 74 | 108 |
| aDS_sDS | CSF_pTau181 | 0.91609756 | 0.87876115 | 0.95343397 | 100 | 123 |
| aDS_sDS | CSF_pTau231 | 0.90833333 | 0.86749511 | 0.94917155 | 98 | 120 |
| aDS_sDS | CSF_pTau205 | 0.89265844 | 0.84422921 | 0.94108768 | 78 | 106 |
| aDS_sDS | CSF_pTau235 | 0.86766529 | 0.81747498 | 0.9178556 | 88 | 110 |
| aDS_sDS | CSF_NTA_Tau | 0.85599694 | 0.80000512 | 0.91198876 | 77 | 102 |
| aDS_sDS | pTau217p_S | 0.95569024 | 0.92809189 | 0.98328858 | 75 | 99 |
| aDS_sDS | pTau181p | 0.88879751 | 0.84450729 | 0.93308774 | 96 | 114 |
| aDS_sDS | pTau231p | 0.88871266 | 0.83963875 | 0.93778657 | 78 | 97 |
| pDS_dDS | CSF_pTau217 | 0.53084416 | 0.41414165 | 0.64754667 | 42 | 66 |
| pDS_dDS | CSF_pTau181 | 0.51916713 | 0.40936276 | 0.6289715 | 49 | 74 |
| pDS_dDS | CSF_pTau231 | 0.50393471 | 0.39212569 | 0.61574373 | 47 | 73 |
| pDS_dDS | CSF_pTau205 | 0.56210131 | 0.44404689 | 0.68015574 | 41 | 65 |
| pDS_dDS | CSF_pTau235 | 0.4803719 | 0.36560054 | 0.59514326 | 44 | 66 |
| pDS_dDS | CSF_NTA_Tau | 0.4891129 | 0.36878628 | 0.60943953 | 40 | 62 |
| pDS_dDS | pTau217p_S | 0.69223986 | 0.58360899 | 0.80087073 | 36 | 63 |
| pDS_dDS | pTau181p | 0.68538961 | 0.58409904 | 0.78668018 | 44 | 70 |
| pDS_dDS | pTau231p | 0.69707207 | 0.59036654 | 0.80377761 | 37 | 60 |
| aDSneg_aDSpos | CSF_pTau217 | 0.85270979 | 0.75878307 | 0.94663651 | 52 | 22 |
| aDSneg_aDSpos | CSF_pTau181 | 0.87210615 | 0.78880111 | 0.9554112 | 77 | 23 |
| aDSneg_aDSpos | CSF_pTau231 | 0.84065315 | 0.73728909 | 0.94401722 | 74 | 24 |
| aDSneg_aDSpos | CSF_pTau205 | 0.89607493 | 0.81676009 | 0.97538977 | 59 | 19 |
| aDSneg_aDSpos | CSF_pTau235 | 0.76033058 | 0.63286812 | 0.88779303 | 66 | 22 |
| aDSneg_aDSpos | CSF_NTA_Tau | 0.88203267 | 0.80598647 | 0.95807887 | 58 | 19 |
| aDSneg_aDSpos | pTau217p_S | 0.9005848 | 0.79265699 | 1 | 57 | 18 |
| aDSneg_aDSpos | pTau181p | 0.74355037 | 0.63104372 | 0.85605701 | 74 | 22 |
| aDSneg_aDSpos | pTau231p | 0.57308468 | 0.41092256 | 0.7352468 | 62 | 16 |
| aDSpos_pDS | CSF_pTau217 | 0.69426407 | 0.56384743 | 0.82468071 | 22 | 42 |
| aDSpos_pDS | CSF_pTau181 | 0.71783496 | 0.59384575 | 0.84182417 | 23 | 49 |
| aDSpos_pDS | CSF_pTau231 | 0.71985816 | 0.59449099 | 0.84522533 | 24 | 47 |
| aDSpos_pDS | CSF_pTau205 | 0.65404365 | 0.51173245 | 0.79635484 | 19 | 41 |
| aDSpos_pDS | CSF_pTau235 | 0.70661157 | 0.5795804 | 0.83364274 | 22 | 44 |
| aDSpos_pDS | CSF_NTA_Tau | 0.62105263 | 0.46987105 | 0.77223421 | 19 | 40 |
| aDSpos_pDS | pTau217p_S | 0.7654321 | 0.63641979 | 0.89444441 | 18 | 36 |
| aDSpos_pDS | pTau181p | 0.7231405 | 0.59327843 | 0.85300256 | 22 | 44 |
| aDSpos_pDS | pTau231p | 0.74831081 | 0.58835593 | 0.90826569 | 16 | 37 |

Abbreviations: AUC, Area Under the Curve. CI, confidence interval. CENT20, 20 centiloids. CENT25, 25 centiloids, CENT30, 30 centiloids, aDS_neg, asymptomatic Down Syndrome amyloid negative. aDS_pos, asymptomatic Down Syndrome amyloid positive. pDS, prodromal Down Syndrome. dDS, dementia Down Syndrome.

## Supplementary Table 4. Diagnostic accuracy of CSF and plasma biomarkers across biological and clinical conditions restricted to participants with all the markers (Sensitivity analysis).

| Comparison | Marker | AUC | CI_lower | CI_upper | Controls | Cases |
| --- | --- | --- | --- | --- | --- | --- |
| ASTATUS | CSF_pTau217 | 0.93875502 | 0.89547403 | 0.98203601 | 24 | 83 |
| ASTATUS | CSF_pTau181 | 0.92344378 | 0.87539844 | 0.97148911 | 24 | 83 |
| ASTATUS | CSF_pTau231 | 0.91817269 | 0.86595738 | 0.970388 | 24 | 83 |
| ASTATUS | CSF_pTau205 | 0.93047189 | 0.88515202 | 0.97579176 | 24 | 83 |
| ASTATUS | CSF_pTau235 | 0.87198795 | 0.80662888 | 0.93734703 | 24 | 83 |
| ASTATUS | CSF_NTA_Tau | 0.87048193 | 0.80140533 | 0.93955852 | 24 | 83 |
| ASTATUS | pTau217p_S | 0.97640562 | 0.95326506 | 0.99954619 | 24 | 83 |
| ASTATUS | pTau181p | 0.86646586 | 0.77886013 | 0.9540716 | 24 | 83 |
| ASTATUS | pTau231p | 0.84312249 | 0.76982239 | 0.91642259 | 24 | 83 |
| TSTATUS | CSF_pTau217 | 0.94013304 | 0.89198044 | 0.98828564 | 41 | 66 |
| TSTATUS | CSF_pTau181 | 1 | 1 | 1 | 41 | 66 |
| TSTATUS | CSF_pTau231 | 0.9563932 | 0.91101388 | 1 | 41 | 66 |
| TSTATUS | CSF_pTau205 | 0.97801183 | 0.9518341 | 1 | 41 | 66 |
| TSTATUS | CSF_pTau235 | 0.96600148 | 0.9307443 | 1 | 41 | 66 |
| TSTATUS | CSF_NTA_Tau | 0.95750185 | 0.91588515 | 0.99911854 | 41 | 66 |
| TSTATUS | pTau217p_S | 0.89209165 | 0.83014531 | 0.95403799 | 41 | 66 |
| TSTATUS | pTau181p | 0.82298596 | 0.73856791 | 0.907404 | 41 | 66 |
| TSTATUS | pTau231p | 0.76995565 | 0.67425095 | 0.86566036 | 41 | 66 |
| CENT20 | CSF_pTau217 | 0.80769231 | 0.56812418 | 1 | 8 | 13 |
| CENT20 | CSF_pTau181 | 0.91346154 | 0.79483912 | 1 | 8 | 13 |
| CENT20 | CSF_pTau231 | 0.78846154 | 0.55086053 | 1 | 8 | 13 |
| CENT20 | CSF_pTau205 | 0.86538462 | 0.68322395 | 1 | 8 | 13 |
| CENT20 | CSF_pTau235 | 0.79807692 | 0.59578995 | 1 | 8 | 13 |
| CENT20 | CSF_NTA_Tau | 0.76923077 | 0.52558512 | 1 | 8 | 13 |
| CENT20 | pTau217p_S | 0.93269231 | 0.82284179 | 1 | 8 | 13 |
| CENT20 | pTau181p | 0.84615385 | 0.6645511 | 1 | 8 | 13 |
| CENT20 | pTau231p | 0.79807692 | 0.57026934 | 1 | 8 | 13 |
| CENT25 | CSF_pTau217 | 0.80769231 | 0.56812418 | 1 | 8 | 13 |
| CENT25 | CSF_pTau181 | 0.91346154 | 0.79483912 | 1 | 8 | 13 |
| CENT25 | CSF_pTau231 | 0.78846154 | 0.55086053 | 1 | 8 | 13 |
| CENT25 | CSF_pTau205 | 0.86538462 | 0.68322395 | 1 | 8 | 13 |
| CENT25 | CSF_pTau235 | 0.79807692 | 0.59578995 | 1 | 8 | 13 |
| CENT25 | CSF_NTA_Tau | 0.76923077 | 0.52558512 | 1 | 8 | 13 |
| CENT25 | pTau217p_S | 0.93269231 | 0.82284179 | 1 | 8 | 13 |
| CENT25 | pTau181p | 0.84615385 | 0.6645511 | 1 | 8 | 13 |
| CENT25 | pTau231p | 0.79807692 | 0.57026934 | 1 | 8 | 13 |
| CENT30 | CSF_pTau217 | 0.77777778 | 0.54796335 | 1 | 9 | 12 |
| CENT30 | CSF_pTau181 | 0.87962963 | 0.73540406 | 1 | 9 | 12 |
| CENT30 | CSF_pTau231 | 0.75 | 0.51809432 | 0.98190568 | 9 | 12 |
| CENT30 | CSF_pTau205 | 0.84259259 | 0.66507596 | 1 | 9 | 12 |
| CENT30 | CSF_pTau235 | 0.76851852 | 0.55892394 | 0.9781131 | 9 | 12 |
| CENT30 | CSF_NTA_Tau | 0.71296296 | 0.46649015 | 0.95943577 | 9 | 12 |
| CENT30 | pTau217p_S | 0.9537037 | 0.86982303 | 1 | 9 | 12 |
| CENT30 | pTau181p | 0.87962963 | 0.72730194 | 1 | 9 | 12 |
| CENT30 | pTau231p | 0.85185185 | 0.6537125 | 1 | 9 | 12 |
| aDS_pDS | CSF_pTau217 | 0.78764479 | 0.67251262 | 0.90277696 | 37 | 28 |
| aDS_pDS | CSF_pTau181 | 0.79826255 | 0.68370867 | 0.91281643 | 37 | 28 |
| aDS_pDS | CSF_pTau231 | 0.7953668 | 0.68805126 | 0.90268233 | 37 | 28 |
| aDS_pDS | CSF_pTau205 | 0.81998069 | 0.71072447 | 0.92923692 | 37 | 28 |
| aDS_pDS | CSF_pTau235 | 0.77027027 | 0.64665285 | 0.89388769 | 37 | 28 |
| aDS_pDS | CSF_NTA_Tau | 0.7519305 | 0.62622412 | 0.87763689 | 37 | 28 |
| aDS_pDS | pTau217p_S | 0.89237452 | 0.81397808 | 0.97077095 | 37 | 28 |
| aDS_pDS | pTau181p | 0.79005792 | 0.67600765 | 0.90410818 | 37 | 28 |
| aDS_pDS | pTau231p | 0.81563707 | 0.70557613 | 0.92569801 | 37 | 28 |
| aDS_dDS | CSF_pTau217 | 0.87323037 | 0.79016385 | 0.9562969 | 37 | 42 |
| aDS_dDS | CSF_pTau181 | 0.89736165 | 0.82410605 | 0.97061725 | 37 | 42 |
| aDS_dDS | CSF_pTau231 | 0.86486486 | 0.77923276 | 0.95049697 | 37 | 42 |
| aDS_dDS | CSF_pTau205 | 0.9047619 | 0.83491545 | 0.97460836 | 37 | 42 |
| aDS_dDS | CSF_pTau235 | 0.85135135 | 0.76367074 | 0.93903197 | 37 | 42 |
| aDS_dDS | CSF_NTA_Tau | 0.83397683 | 0.7430845 | 0.92486917 | 37 | 42 |
| aDS_dDS | pTau217p_S | 0.97683398 | 0.94918225 | 1 | 37 | 42 |
| aDS_dDS | pTau181p | 0.94337194 | 0.8931709 | 0.99357299 | 37 | 42 |
| aDS_dDS | pTau231p | 0.94047619 | 0.88441499 | 0.99653739 | 37 | 42 |
| aDS_sDS | CSF_pTau217 | 0.83899614 | 0.75914945 | 0.91884283 | 37 | 70 |
| aDS_sDS | CSF_pTau181 | 0.85772201 | 0.78226012 | 0.9331839 | 37 | 70 |
| aDS_sDS | CSF_pTau231 | 0.83706564 | 0.75670802 | 0.91742326 | 37 | 70 |
| aDS_sDS | CSF_pTau205 | 0.87084942 | 0.79779288 | 0.94390596 | 37 | 70 |
| aDS_sDS | CSF_pTau235 | 0.81891892 | 0.73782056 | 0.90001727 | 37 | 70 |
| aDS_sDS | CSF_NTA_Tau | 0.8011583 | 0.71370083 | 0.88861577 | 37 | 70 |
| aDS_sDS | pTau217p_S | 0.94305019 | 0.90146428 | 0.98463611 | 37 | 70 |
| aDS_sDS | pTau181p | 0.88204633 | 0.81377026 | 0.9503224 | 37 | 70 |
| aDS_sDS | pTau231p | 0.89054054 | 0.82735303 | 0.95372805 | 37 | 70 |
| pDS_dDS | CSF_pTau217 | 0.5795068 | 0.43795805 | 0.72105555 | 28 | 42 |
| pDS_dDS | CSF_pTau181 | 0.5795068 | 0.43300337 | 0.72601024 | 28 | 42 |
| pDS_dDS | CSF_pTau231 | 0.56377551 | 0.41670487 | 0.71084615 | 28 | 42 |
| pDS_dDS | CSF_pTau205 | 0.57653061 | 0.4303854 | 0.72267583 | 28 | 42 |
| pDS_dDS | CSF_pTau235 | 0.54591837 | 0.40001595 | 0.69182078 | 28 | 42 |
| pDS_dDS | CSF_NTA_Tau | 0.46598639 | 0.3216745 | 0.61029829 | 28 | 42 |
| pDS_dDS | pTau217p_S | 0.73596939 | 0.61391575 | 0.85802302 | 28 | 42 |
| pDS_dDS | pTau181p | 0.7329932 | 0.60902178 | 0.85696461 | 28 | 42 |
| pDS_dDS | pTau231p | 0.71130952 | 0.58925943 | 0.83335961 | 28 | 42 |
| aDSneg_aDSpos | CSF_pTau217 | 0.97115385 | 0.92905747 | 1 | 24 | 13 |
| aDSneg_aDSpos | CSF_pTau181 | 0.85576923 | 0.73040166 | 0.9811368 | 24 | 13 |
| aDSneg_aDSpos | CSF_pTau231 | 0.87179487 | 0.75827706 | 0.98531268 | 24 | 13 |
| aDSneg_aDSpos | CSF_pTau205 | 0.84294872 | 0.70634997 | 0.97954746 | 24 | 13 |
| aDSneg_aDSpos | CSF_pTau235 | 0.79487179 | 0.64239753 | 0.94734606 | 24 | 13 |
| aDSneg_aDSpos | CSF_NTA_Tau | 0.80769231 | 0.66542667 | 0.94995795 | 24 | 13 |
| aDSneg_aDSpos | pTau217p_S | 0.94070513 | 0.86750437 | 1 | 24 | 13 |
| aDSneg_aDSpos | pTau181p | 0.71955128 | 0.55489006 | 0.8842125 | 24 | 13 |
| aDSneg_aDSpos | pTau231p | 0.48076923 | 0.28226818 | 0.67927028 | 24 | 13 |
| aDSpos_pDS | CSF_pTau217 | 0.58791209 | 0.41059661 | 0.76522757 | 13 | 28 |
| aDSpos_pDS | CSF_pTau181 | 0.6510989 | 0.48107641 | 0.8211214 | 13 | 28 |
| aDSpos_pDS | CSF_pTau231 | 0.60714286 | 0.42615166 | 0.78813406 | 13 | 28 |
| aDSpos_pDS | CSF_pTau205 | 0.67307692 | 0.50142958 | 0.84472427 | 13 | 28 |
| aDSpos_pDS | CSF_pTau235 | 0.6510989 | 0.48332867 | 0.81886913 | 13 | 28 |
| aDSpos_pDS | CSF_NTA_Tau | 0.6043956 | 0.42215537 | 0.78663583 | 13 | 28 |
| aDSpos_pDS | pTau217p_S | 0.75686813 | 0.60116393 | 0.91257234 | 13 | 28 |
| aDSpos_pDS | pTau181p | 0.73901099 | 0.58217213 | 0.89584985 | 13 | 28 |
| aDSpos_pDS | pTau231p | 0.79395604 | 0.63968167 | 0.94823042 | 13 | 28 |

Abbreviations: AUC, Area Under the Curve. CI, confidence interval. CENT20, 20 centiloids. CENT25, 25 centiloids, CENT30, 30 centiloids, aDS_neg, asymptomatic Down Syndrome amyloid negative. aDS_pos, asymptomatic Down Syndrome amyloid positive. pDS, prodromal Down Syndrome. dDS, dementia Down Syndrome.

# Supplementary Figures


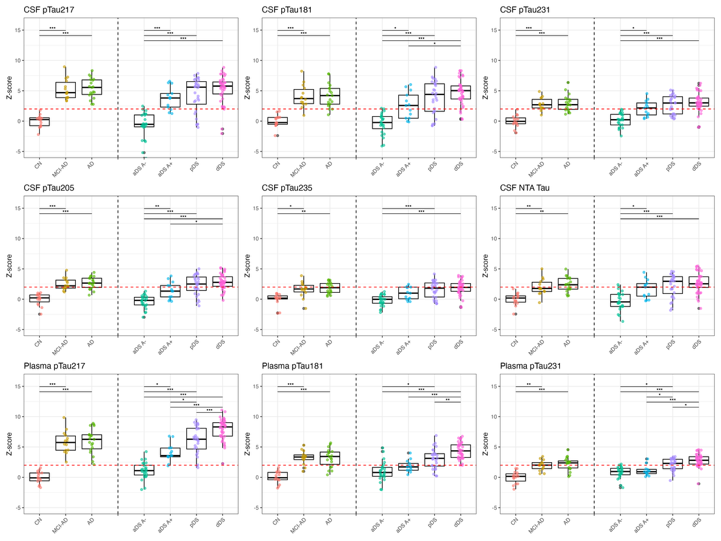


## Supplementary Figure 1. Concentrations of tau markers in CSF and plasma

The figure shows different concentrations of Tau markers in CSF and plasma. Biomarkers were first log transformed and then Z-scores were calculated from age-matched CN. We can observe the stepwise increase in all plasma markers in symptomatic stages of DS. This pattern is not present in sporadic AD, neither in any CSF Tau marker in both populations, where plateaus in mild symptomatic phases.

Abbreviations: CN, cognitively normal. MCI-AD, mild cognitive impairment Alzheimer’s Disease. AD, Alzheimer’s Disease. aDS A-, asymptomatic Down Syndrome CSF Amyloid negative. aDS A+, asymptomatic Down Syndrome CSF Amyloid positive. pDS, prodromal Down Syndrome. dDS, dementia Down Syndrome.

Supplementary Figure 2. Estimated trajectories of tau biomarkers in Down syndrome (only participants with all Tau markers available)

We include only participants with all biomarkers. CSF and plasma Tau biomarkers exhibit a uniform pattern of abnormality across age in DS. In Down syndrome, all these biomarkers display a similar age of abnormality onset. As there are included only 15 CN to normalize, confidence intervals are thicker than those shown in the LOESS in the main text. To characterize age-related changes in tau biomarkers along the Down syndrome (DS)–Alzheimer's continuum, we applied locally estimated scatterplot smoothing (LOESS) models to the log-transformed biomarker concentrations. For each tau biomarker, we compared the log-transformed concentrations in individuals with DS against those of age-matched cognitively normal (CN) euploid controls across the lifespan. LOESS fits were generated with locally weighted polynomial regressions, using a smoothing parameter (α) of 0.75. The shaded bands around each LOESS curve represent the 95% confidence interval of the fit. The point of divergence between DS and control trajectories was defined as the age at which these confidence bands no longer overlapped, indicating the onset of statistically relevant deviation from control biomarker levels. The horizontal black dashed lines indicate the cutoffs corresponding to ±2 standard deviations from the mean of the CN reference group, used as the threshold for biomarker abnormality. Vertical dashed lines mark established milestones along the DS–AD continuum (amyloid pseudonormality, CSF amyloid positivity, and amyloid PET positivity). For plasma biomarkers, a secondary x-axis displays the estimated years to symptom onset. Abbreviations: DS, Down Syndrome; CN, cognitively normal.

##
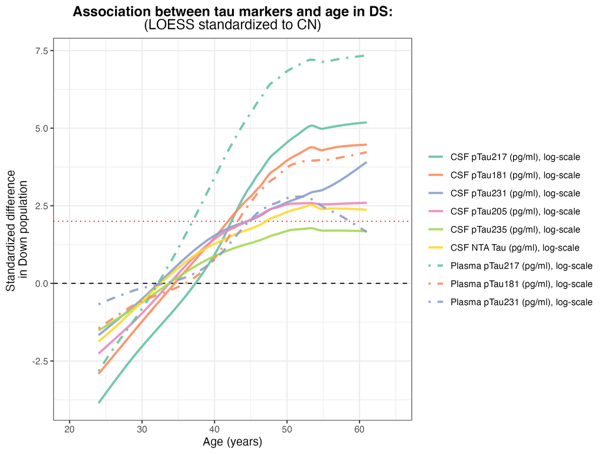
Supplementary Figure 3. Estimated trajectories of tau biomarkers in Down syndrome and their association with age (only participants with all Tau markers available)

We only include participants with all the markers. Continuous lines represent CSF biomarkers, while dashed lines indicate plasma biomarkers. Corresponding plasma and CSF markers are depicted in matching colors. The cutoff of two standard deviations is indicated by a red dashed line. No significant temporal differences were observed between different tau isoforms in CSF in Down syndrome, suggesting a more stretched timespan of Tau accumulation at the beginning. All markers trend to increase from the 3^rd^ decade. In a range of 10 years (4^th^ decade to 5^th^ decade) all CSF markers reach +2SD from normality, except for plasma pTau217 that reaches abnormality before 4^th^ decade. Then, we could artificially differentiate 3 groups, the earliest one would be pTau217 in plasma. The second group would be CSF and plasma pTau181 and CSF pTau217, and the last one all the other markers.

## Supplementary Figure 4. Estimated trajectories of tau biomarkers in symptomatic stages in Down syndrome (a) and sporadic AD (b). Only participants with all Tau markers available.

Continuous lines represent CSF biomarkers, while dashed lines indicate plasma biomarkers. Corresponding plasma and CSF markers are depicted in matching colors. The cutoff of two standard deviations is indicated by a red dashed line. During symptomatic phases, tau biomarkers in Down syndrome slow their rate of increase with age in both CSF and plasma. In contrast, in euploid individuals, CSF tau levels tend to converge over time, reducing the gap between cognitively normal (CN) individuals and those with sporadic AD.
